# NHS funding for dental undergraduate human disease teaching in the UK: a 20-year review

**DOI:** 10.1038/s41415-022-5099-4

**Published:** 2022-10-28

**Authors:** Philip A. Atkin, Benjamin A. Jones

**Affiliations:** Cardiff University School of Dentistry, Cardiff, UK

## Abstract

**Introduction ** This study identifies funding of NHS services supporting dental students' teaching over the last 20 years, focusing on human disease (HD) teaching.

**Aims**To identify NHS funding for education in UK dental schools following publication of the 1997 General Dental Council curriculum introducing specific funding for HD teaching and the years 2015/16 to 2019/20.

**Material and methods**Searches of the medical literature, grey literature (government and regulatory authority reports, legislative articles) and freedom of information requests to hospitals helping to deliver teaching.

**Results**There are few publications describing current funding of dental undergraduate teaching. Freedom of information requests gave data for NHS hospital allocations for teaching both clinical dentistry and HD. HD funding has dropped by £2 million in five years.

**Conclusions**NHS Trusts linked to dental schools receive monies to deliver teaching and offset costs of accommodating students. Tracking these funds over 20 years has seen some schools lose up to £1 million of HD funding and some lose it all. Greater transparency regarding funding for HD delivery would help improve teaching. Increasing numbers of older patients, with a greater chronic disease burden who are retaining teeth into later life, need graduating dentists with good medical knowledge to deliver safe care.

**Supplementary Information:**

Zusatzmaterial online: Zu diesem Beitrag sind unter 10.1038/s41415-022-5099-4 für autorisierte Leser zusätzliche Dateien abrufbar.

## Key points

- Graduating dental students need a sound understanding of medicine relevant to the safe care of their patients.
- Foundation dentist (FD) trainers in UK general dental practice need to know their FD has the knowledge to safely care for their patients.
- As taxpayers, the readers need to know that NHS funds allocated for dental student undergraduate teaching are directed appropriately, especially for human disease funds into general hospital budgets.
- General Dental Council (GDC) staff charged with revising and developing curricula need to be aware that human disease was previously recognised by the GDC as a key element of undegraduate teaching and needs to remain so, with emphasis in future curriculums.

**Supplementary Information:**

Zusatzmaterial online: Zu diesem Beitrag sind unter 10.1038/s41415-022-5099-4 für autorisierte Leser zusätzliche Dateien abrufbar.

## Introduction

With an ageing UK population living with chronic disease and using more drugs to manage their disorders,^1^ a sound knowledge of medicine in our graduating dentists has never been more important, hence the need for dedicated teaching in human disease (HD) in the undergraduate curriculum.

Accommodating medical and dental undergraduates into NHS clinics for teaching means that fewer patients are seen, displaced patients are moved to additional service clinics and the NHS provider is reimbursed for that additional cost by central government. In England and Wales, this funding is known as the Service Increment for Teaching (SIFT).^2^ In Northern Ireland, the additional costs to the NHS for teaching both medical and dental students in NHS clinics is called the Supplement for Undergraduate Medical and Dental Education (SUMDE) and in Scotland, the Additional Costs for Teaching (Dental) or ACT(D). In Scotland, the element of funding for dental students in medicine, that is, the HD teaching element, comes from the ACT for Medicine (ACTM) budget and is called ACTM-Dental. These SIFT funds apply in both medical and dental undergraduate teaching in NHS clinics and the monies are directed to the NHS to allow them to provide the additional personnel and administration costs associated with teaching undergraduates in service clinics. Teaching dental students in NHS service clinics in dental hospitals has dental SIFT (dSIFT) funding directed for the additional costs incurred. NHS SIFT funds were first introduced in 1976.^3^ In 1989, SIFT was renamed SIFTR, with the 'R' representing elements of research funding.^4^The original name of SIFT has stuck, however, and is more commonly used.

Two examples of how the additional costs for teaching may be recognised and be reimbursed to the NHS are given in Box 1.

In 1997, the UK General Dental Council (GDC) identified in their First Five Years (FFY) curriculum^5^ that the teaching of medicine and surgery/human disease needed some specific direction and described where and how dental students should attend clinics for teaching by NHS colleagues. The FFY curriculum said dental students should attend 'in-patient and out-patient medical and surgical departments or in specialist clinics situated in teaching or district general hospitals' (paragraph 75).

The GDC FFY curriculum also described the funding stream to go to the NHS to offset the additional costs incurred by the NHS for accepting dental students in dental hospitals 'National Purchasing Unit for Dental SIFT which purchases services from providers necessary for undergraduate dental education on behalf of dental deans or equivalent persons' (paragraph 50).

In relation to the new and more detailed focus on HD and for the first and only time, the GDC curriculum also said 'part of the undergraduate dental curriculum must be devoted to instruction in medicine and surgery (human disease) and to attendance at accident and emergency departments. A Trust, usually the host Trust, is provided with specific funding to supply the facilities and staff for this part of the curriculum and it must be used for that purpose in agreement with the dental dean or equivalent person' (paragraph 55).

This 'specific funding' stream is known as Medical for Dental SIFT (M4D SIFT) and is dedicated to dental student human diseases teaching.

Many schools adapted to the new direction for HD teaching coming from the FFY curriculum by changing the undergraduate programme so students would attend medical and surgical clinics and ward rounds, and to attend sessions in hospital emergency departments, the timetabling and content supervised by a dedicated liaison in the hospital working alongside a dental school staff member. Changes in arrangements for inpatient care and later versions of the GDC curriculum with less defined direction for HD teaching mean many schools no longer send dental students into district hospitals for HD teaching but the funding streams persist.

It is possible that in 1997, the GDC was prescient enough to recognise that as the decades rolled by, the population, and therefore dental patients, would be living longer, retaining more of their natural dentition, suffer more chronic disease and need an increasingly long list of medications to manage these disorders and that future dental graduates needed to be fully equipped with the medical knowledge to safely care for these patients. Indeed, the speciality of special care dentistry came about in response to the increasing number of patients who required routine dental care but their medical complexity meant they may not be suitable for treatment in a typical high-street dental practice.^6^

In many UK dental schools, the dean is also the director of NHS service in the dental hospital; in others, the dean and NHS clinical director are separate roles but typically one building serves both functions. The dental SIFT money flowing into the NHS dental hospital might be managed by the dental school dean and NHS clinical director to benefit dental undergraduate teaching within the NHS dental service. In the case of dental student HD teaching, the M4D SIFT money flows into the linked general hospital and the departments of medicine, surgery and emergency medicine. The finance director of the NHS Hospital Trust may be the only person with a detailed view of where these M4D SIFT funds are directed. The head of the dental hospital and school may have some knowledge and possibly little control over where these funds go and how they support HD teaching for dental students.

***Box 1***Examples of the additional costs to the NHS of teaching undergraduates **Scenario 1**

An NHS consultant cardiologist has two outpatient clinics with 20 patients in attendance at each clinic. The consultant is asked to accept six dental students to each clinic for teaching as part of the HD programme and reduce patient numbers by half to facilitate this. A second cardiologist provides care for the 20 displaced patients in an additional clinic needing time, clinic space, nursing and administrative support. The additional clinic is funded from M4D SIFT monies.

**Scenario 2**

An NHS consultant dermatologist has an outpatient clinic with 20 patients. Instead of attending the clinic, they are asked to give a half-day symposium of teaching to dental students as part of their HD programme. A second consultant dermatologist is needed to provide the care for those patients whose consultant has been taken from a service clinic to provide dental undergraduate HD teaching. The replacement dermatologist is funded from M4D SIFT monies.

## Aims and objectives

Without funding, the education and training of dental undergraduates would be impossible. The purpose of this study is to identify monies allocated to the UK NHS to support dental student teaching and in particular, the specific funding allocated to NHS hospital trusts to help deliver dental undergraduate HD teaching in UK dental schools. Beginning with the UK GDC FFY curriculum in 1997 and GDC reviews in their dental school inspections in 2003-2005,^7^there was increased focus on the content, delivery and funding of HD teaching. We sought to establish what funding was being provided to dental hospitals directly and to the linked general hospitals for HD teaching using these reports, the value and allocation of these same funds in the most recent five years and to determine what changes, if any, had occurred over the years.

## Materials and methods

We began with a literature review searching for mentions of SIFT and M4D SIFT in relation to funding of undergraduate medical and dental education. The search was conducted using Medline^8^ for publications in the medical literature and an internet search engine^9^ to identify wider information sources. In Medline, we used the following Medical Subject Headings terms: SIFT; Service Increment for Teaching; MPET; Multi Professional Education and Training; Dental Education; Medical Education; England and Wales, which were paired with the Boolean operators "AND" and "OR" to create the search criteria. Secondary sources were accumulated by assessing the reference lists for each of the journal articles located to ensure no key entries had been missed. Internet searches helped to identify 'grey literature' - materials and research produced in a non-commercial form, including government publications and reports, legislative articles and reports by regulatory authorities, such as the GDC. Searches identified documents regarding the general development of SIFT funding in medical and dental schools, the specific SIFT monies allocated to medicine (mSIFT) and also dental SIFT monies (dSIFT and M4D SIFT). Since these literature searches revealed little detailed data on current dSIFT and M4D SIFT, freedom of information (FoI) requests were submitted to the NHS Trusts and organisations linked to dental teaching hospitals and schools in the four UK countries using a similar approach to that used by the British Medical Association for their 2007 SIFT funding report.^10^FoI requests were made using two methods: a web-based FoI request tool, which tracked requests and alerted when responses were received and also allowed publication of responses,^11^ and direct requests to individual NHS Hospital Trusts using their FoI online templates or dedicated FoI email addresses.

## Results

### Papers in the published medical literature

The initial medical literature search for references to SIFT (including mSIFT, dSIFT and M4D SIFT) found just six papers and from examining these, two main themes emerged. The first theme was that SIFT allocation is arbitrary and not evidence-based.^2^^,^^12^^,^^13^^,^^14^^,^^15^The second, that allocation of SIFT should be based on evidence and also the quality of learning delivered.^12^^,^^14^^,^^16^

### Papers published in the 'grey literature'

A search of relevant articles revealed 55 publications in an initial screen. After selecting for relevance and full-text availability, nine remained.^10^^,^^17^^,^^18^^,^^19^^,^^20^^,^^21^^,^^22^^,^^23^ Three common themes from these publications were identified: 1) lack of transparency in allocation of funds; 2) SIFT not always used for its intended purpose; and 3) SIFT allocation appears to be arbitrary, as shown in online Supplementary Table 1.

### Freedom of information data

The principal source of recent information on SIFT was from FoI requests to the NHS Trusts which partner with UK dental hospitals and schools. The trusts were asked to give figures for dSIFT funding they received for the years 2015/16 to 2019/20, the M4D SIFT for the same years, and dSIFT and M4D SIFT equivalents in Scotland and Northern Ireland. Online Supplementary Table 2 shows the responses from the NHS Trusts in England, Wales, Scotland and Northern Ireland with linked dental schools, as well as data from the GDC inspection reports of dental schools (2003-2005).^7^

There are 16 undergraduate dental schools in the UK. Three dental schools, (Aberdeen, Peninsula and University of Central Lancashire) are newer schools established between 2006 and 2009. These schools therefore post-date the instructions from the 1997 GDC FFY curriculum regarding the funding and delivery of HD teaching and so may not have had historical M4D SIFT funding streams. However, FoI requests did produce data on dSIFT and M4D SIFT in these schools.

## Discussion

Examination of publications in the medical literature found relatively few papers discussing or describing SIFT and most of those are around 20-30 years old. Although SIFT was introduced in 1976, most of the publications appear between 1990 and 2001. Themes revealed through examining the published literature included: SIFT allocation is arbitrary and not evidence-based; new methods of allocating SIFT were proposed; allocations should be evidence-based; and that allocation of SIFT monies should be based on evidence of, and quality of, learning. It is likely that the lack of transparency must have made data collection for journal publications difficult and evaluation of fair allocation of funds near impossible and the same applies today.

In the grey literature, the mentions of SIFT began in 2002. M4D SIFT starts with the GDC dental school inspection reports from 2003-2005^7^ when the effects of the GDC 1997 FFY instructions on the allocation of a new M4D SIFT for HD teaching and assessment were reviewed. Similar themes are revealed in the grey literature: lack of transparency, inappropriate or unclear usage of funds and the apparently arbitrary allocation of funds.

The historical GDC school inspection reports for many schools include detail on dSIFT and M4D SIFT allocations. Some schools were unwilling or unable to give detail. The inability to account for SIFT monies was noted by the GDC inspections from 2003-2005.^7^ For the ten schools that did give SIFT data, the figures are interesting. M4D SIFT figures ranged from as little as £110,000 to as much as £1.0M. While different dental schools may have had different numbers of students enrolled, the figures would not differ by as much as a factor of nearly ten. Seven schools had M4D SIFT funding greater than £600,000, with two reporting sums of £300,000 or less. An interesting figure is the percentage (to the nearest 0.5%) of M4D SIFT as a proportion of the total SIFT funding (M4D SIFT plus dSIFT) which ranges from as little as 3% to as much as 15.5% in the different schools: differences greater than a factor of five. If HD teaching is given the same prominence in each school, then the funding allocated to the subject ought to be broadly similar also.

In relation to more recent M4D SIFT and dSIFT figures, online searches of NHS trust accounts were fruitless, with dental-related SIFT figures likely too small to feature in annual reports. For this reason, FoI requests proved the most useful sources of current information and show some trends in the five-year data that was requested. In some schools, the M4D SIFT had been explicitly incorporated into the total dSIFT or it was not separately accounted for. In at least one school, nearly £2 million in M4D SIFT was described in their 2015/16 figures but apparently disappeared altogether in the following years. The corresponding dSIFT in this school did not increase at all, so it is unclear where the M4D SIFT funding went or how the HD teaching programme would be supported thereafter.

In two of the three newer (post-FFY 1997 curriculum) dental hospitals and schools, there was either no dSIFT at all, or for those that did have a SIFT funding stream, there was no separate M4D SIFT. In the case of the third school, there are three linked NHS trusts and all three have dSIFT allocations, but only one had a M4D SIFT allocation.

Five dental hospitals have had their dSIFT close to doubled between their 2003-2005 GDC report and the 2015/16 to 2019/20 data, whereas others had changes in the few thousands of pounds either upwards or downwards in the years since the GDC inspections.

In relation to the recent five-year data from FoI requests, the dSIFT and M4D SIFT monies in most schools that declared separate dSIFT and M4D SIFT streams have remained more or less static. However, an interesting finding is the huge variation in the percentage of M4D SIFT in relation to dSIFT in these schools. In the most recent figures supplied, the highest is 12% of the total SIFT figure and the lowest, about 1%. The highest allocation of M4D SIFT in 2015/2016 was £1.9 million and the lowest was £0 where there was no separate allocation. For the most recent M4D SIFT figures (2019/2020), the sums range from £1 million to £59,000, where M4D SIFT was a separate allocation. The total declared M4D SIFT monies in 2015/16 were £7.2 million and in 2019/20, £5.3 million (a drop of nearly £2 million across the UK schools). This is a significant sum and given the increasing need for graduating dental students to have a solid grounding in HD knowledge and understanding, but delivered on a reducing teaching budget, this ought to be of concern to students, teachers and patients alike.

Limitations or weaknesses of this study relate to the paucity of SIFT-related literature in the published medical literature. Mostly references are made in reports from interested organisations and are found through grey literature searches which may be incomplete. We also relied on NHS Trust finance departments to supply accurate data which cannot be cross-checked against published NHS Trust annual finance reports, since SIFT is never mentioned. Strengths of this study relate to the thorough methodology of medical literature and grey literature searches and the identification of references in these documents to identify previously unidentified resources.

## Summary

Despite the importance of medical knowledge in our graduating dental students for the safe delivery of dental care for a population with increasing age, illness and chronic disease^1^^,^^24^ and needing more dentistry (retaining natural dentition into older age),^25^the dedicated funding streams for delivering this HD teaching in many dental hospitals and schools are reducing, or have seemingly disappeared altogether, according to the figures supplied following FoI requests (online Supplementary Table 2). Examining the medical literature and the grey literature reveals there is often a lack of clarity in the allocation and use of SIFT monies and this may make it difficult for dental school deans to accurately direct NHS monies allocated specifically to support HD teaching. The GDC may need to consider revising future undergraduate curricula to help guide dental schools and the linked hospital funding streams, as they did for the first and only time in their 1997 FFY curriculum document, or include HD funding as a dedicated inspection item in future dental school inspections. There needs to be greater transparency of M4D SIFT monies and ideally, a greater control of these monies by HD teaching leads, to the benefit of dental undergraduate students and ultimately, their future patients.

## Supplementary Information

Supplementary tables (PDF 148KB)
