# Supplementary material for: NHS funding for dental undergraduate human disease teaching in the UK: a 20-year review
Source: Br Dent J. 2022 Oct 28;233(8):675–8. doi: 10.1038/s41415-022-5099-4 (PMC9616713; doi:10.1038/s41415-022-5099-4)
Supplement: Supplementary file 1 — Supplementary tables (PDF 148KB) [file 41415_2022_5099_MOESM1_ESM.pdf]

Table 1 SIFT Themes in the grey literature

Theme 1: Lack of transparency	
Document	***Example***
Dept. of Health 2002[17]	Transparency – education commissioners and providers should be able to account fully for their use of funding. Not all education and training funding can be readily tracked - SIFT for example in large measure supports service provision in teaching hospitals rather than education and training direct.<br>Medical SIFT in particular lacks transparency.
BMA 2007[18]	Concerns have been raised that funding flows for medical education are not transparent.<br>The Department of Health acknowledges that they do not know how SIFT is used in most recipient teaching hospitals.<br>It has been acknowledged that in practice it is often difficult to know where NHS clinical services end and university teaching begins.<br>Trusts were asked to account for how the SIFT allocation had been spent in each of the last five years. Around half of respondents (10/23) could not account for how their SIFT funding had been spent over the last five years.
GDC Liverpool 2005[19]	The School is involved in the allocation of these funds through the Dean's role as an adviser to the workforce finance director of the SHA. We believe there should be greater transparency in the allocation of this funding stream.
GDC Leeds 2003[20]	In 2002/3 £135k of the allocation of approximately £750k was received by the Dental School but the basis for the allocation of the remainder was not clear. The School should seek greater transparency in the future allocation of these funds. West Yorkshire WDC should ensure that allocations of Medical SIFT for Dental Students are transparent and reflect the requirements of the Dental School.
GDC Bristol 2003[21]	The Human Disease course for dental undergraduate students is supported by HEFCE and Medical for Dental SIFT funds. We understand that greater transparency of Medical for Dental SIFT allocations would be welcomed by the Dental School.
Dept. of Health 2012[22]	HEE will be responsible for developing a more transparent allocations policy for distributing funding to LETBs.<br>HEE's role will be to ensure greater transparency in the education and training investments employers make in their workforce.
Theme 2: SIFT is not always used for its intended purpose and specific allocation is rarely documented	
Document	***Example***
Dept. of Health 2002[17]	… education commissioners and providers should be able to account fully for their use of funding. Not all education and training funding can be readily tracked - SIFT for example in large measure supports service provision in teaching hospitals rather than education and training direct.

	Medical for dental funding is not always used fairly at field level for the teaching of dental undergraduates.<br>Within the existing budget envelopes, it is hard to redistribute facilities funding into new teaching settings, particularly in the community, without financially destabilising existing centres.
BMA 2007[18]	Few respondents appear to have rigorous auditing or governance structures in place with regard to SIFT funding.
GDC Leeds 2003[20]	… oversight of Medical Service Increment For Teaching for Dental Students monies. In 2002/3 £135k of the allocation of approximately £750k was received by the Dental School but the basis for the allocation of the remainder was not clear.
GDC Bristol 2003[21]	Proper disbursement (…) of the Medical for Dental SIFT allocation must be achieved.<br>The teaching of the management of medical emergencies, including cardiopulmonary resuscitation (CPR), occurs throughout the programme and includes sessions in the UBHT. Medical Clinical Skills Unit using resuscitation simulators. We were told this resource is expensive and costs £500 per half day. It is not clear how this part of the course is supported financially and whether it is covered by the Medical for Dental SIFT allocation.
Steele & Kay 2010[25]	SIFT: In nearly all cases, the Trust receives and handles this money in very different ways depending on local organisation. In some cases the educational provider has almost no say over how this is spent.
Theme 3: SIFT allocation is arbitrary and not evidence-based	
Document	***Example***
Dept. of Health 2002[17]	Funding flows: Whilst NHS funding, allocation, and expenditure is more opaque than HEFCE funding, the Department of Health acknowledges that they have very little idea of how the money allocated to SHAs for medical SIFT is spent. (SHA = Strategic Health Authority).<br>SIFT unevenly distributed on historic principles – mainly as a support for service and weighted towards London teaching hospitals.<br>Rebase placement support to identify clearly the elements that do and do not support education.
Dept. of Health 2013[23]	The variation in levels of funding paid for undergraduate medical education has created an inequity between providers, with those receiving higher sums receiving an unfair advantage over those receiving lower sums. There is little evidence that those receiving larger sums of money are providing placements of a higher quality.
NHS Commissioning Board 2013[24]	Dental schools: SIFT allocations are based on historical numbers of students already in training

Table 2 SIFT figures from GDC school inspection reports (2002–2005) and FoI data for dSIFT and Medical for Dental SIFT (2015–2020)

Dental School /University	***NHS Trust(s)***	***SIFT (or equivalent)***	***GDC Inspection Report data***	***FoI SIFT Responses***				
England & Wales			2003–5	15/16	16/17	17/18	18/19	19/20
Barts & The London, London	Barts Health NHS	dSIFT	£5.717M	£11,214,374	£11,134,106	£10,911,630	£10,911,630	£10,911,630
		MforD SIFT	£967K	£1,982,581	0	0	0	0
		% of total SIFT	14.5%	14.8%	-	-	-	-
Birmingham	Birmingham Community Healthcare NHS	dSIFT	Not stated	£8,999,000	£8,999,000	£8,999,000	£8,893,000	£9,015,000
		MforD SIFT	Not stated	£679,000	£680,000	£681,000	£727,000	£827,000
		% of total SIFT	-	7%	7%	7%	7.5%	8.5%
Bristol	University Hospitals Bristol NHS	dSIFT	Not stated	£9,893,000	£9,364,000	£8,465,000	£8,522,000	£8,861,000
		MforD SIFT	Not stated	£930 - £950,000	£930 - £950,000	£930 - £950,000	£930 - £950,000	£930 - £950,000
		% of total SIFT	-	8.5%	9%	10%	10%	9.5%
Cardiff	Cardiff & Vale UHB NHS	dSIFT	£7.605M	£12,506,000	£12,506,000	£12,506,000	£12,506,000	£12,506,000
		MforD SIFT	£300K	£1,003,000	£1,003,000	£1,003,000	£1,003,000	£1,003,000
		% of total SIFT	4.0%	7.5%	7.5%	7.5%	7.5%	7.5%
King's College, London (x2 sites & x2 NHS Trusts) (King's – London SE5)	Kings College Hospital NHS	dSIFT	Not stated	£4,905,869	£3,853,050	£3,777,060	£3,777,060	£3,777,060
		MforD SIFT	Not stated	"Consolidated into dSIFT prior to 2015"				
		% of total SIFT	-	-	-	-	-	-
	Guys & St Thomas's NHS	dSIFT	Not stated	£18,521,066	£19,308,135	£18,922,330	£18,922,330	£18,922,330
		MforD SIFT	Not stated	0	0	0	0	£111,090
		% of total SIFT	-	-	-	-	-	0.6%

(Guys – London SE1)								
Leeds	Leeds Teaching Hospitals NHS	dSIFT	Not stated	£10,324,347	£10,013,480	£9,869,928	£9,869,929	£9,869,929
		MforD SIFT	£750K	"these are deemed to cover any medical for dental SIFT"				
Liverpool	Royal Liverpool & Broadgreen NHS University Hospitals	dSIFT	Not stated	£9,047,251	£9,047,251	£9,047,251	£9,047,251	£9,047,251
		MforD SIFT	£0.9m	£561,121	£561,972	£548,028	£548,028	£548,028
		% of total SIFT	-	6%	6%	6%	6%	6%
Manchester	Manchester University NHS	dSIFT	£4.117M	£8,783,221	£8,783,220	£8,783,220	£8,783,221	£8,783,221
		MforD SIFT	£765K	£862,591	£862,591	£862,590	£862,591	£862,590
		% of total SIFT	15.5%	6%	6%	6%	6%	6%
Sheffield	Sheffield Teaching Hospitals NHS	dSIFT	£4.951M	£9,557,500	£9,354,700	£9,202,600	£9,202,600	£9,202,600
		MforD SIFT	£745K	"There are no specific amounts allocated for medical for dental SIFT or records of the distribution between medical and dental D SIFT"				
		% of total SIFT	13.0%	-	-	-	-	-
Newcastle	Newcastle upon Tyne Hospitals NHS	dSIFT	£5.719M	£9,247,000	£9,247,000	£9,247,000	£9,247,000	£9,247,000
		MforD SIFT	£600K	0	0	0	0	0
		% of total SIFT	9.5%	-	-	-	-	-
Peninsula, Plymouth	University Hospitals Plymouth NHS	dSIFT	Established 2006	0	0	0	0	0
		MforD SIFT		0	0	0	0	0
University of Central Lancashire (UCLan)	Blackpool Teaching Hospitals NHS	dSIFT	Established 2007	£1,599,244	£1,537,267	£1,545,640	£1,538,640	£1,615,272
		MforD SIFT		0	0	0	0	0
	Lancashire & South	dSIFT	Established 2007	£769,320	£769,320	£769,320	£769,320	£769,320
		MforD SIFT		0	0	0	0	0

(x3 sites, x3 NHS Trusts)	Cumbria NHS							
	North Cumbria Integrated Care NHS	dSIFT	Established 2007	£769,000	£769,000	£769,000	£769,000	£769,000
		MforD SIFT		£65,000	£65,000	£95,000	£104,000	£102,000
		% of total SIFT		8.0%	8.0%	11.0%	12%	12%
		dSIFT	TOTAL	£3,137,564	£3,075,587	£3,083,960	£3,076,960	£3,153,592
		MforD SIFT	TOTAL	£65,000	£65,000	£95,000	£104,000	£102,000
		% of total SIFT		2.0%	2.0%	3.0%	3.0%	3.0%
Scotland			2003–5	15/16	16/17	17/18	18/19	19/20
Aberdeen	Grampian NHS	ACT(D)	Established 2009	£3,146,000	£3,147,000	£3,229,000	£3,309,000	£2,700,000
		ACT(M) for D		No figures supplied				
Dundee	Tayside NHS	ACT(D)	£3.58M	£4,982,816	£4,982,816	£5,317,074	£5,177,499	£5,177,499
		ACT(M) for D	£110,000	£468,089	£463,972	£447,894	£447,894	£447,894
		% of total ACT	3.1%	9.5%	9.5%	8.0%	9.0%	9.0%
Glasgow	Greater Glasgow & Clyde NHS	ACT(D)	Not stated	£8,571,932	£8,571,932	£8,657,651	£9,062,106	£9,062,106
		ACT(M) for D	£1.0M	£648,868	£606,495	£612,560	£612,560	£612,560
		% of total ACT	-	8.0%	7.0%	7.0%	7.0%	7.0%
Northern Ireland			2003–5	15/16	16/17	17/18	18/19	19/20
Belfast, Queens	Belfast Health & Social Care NHS	SUMDE	£4.0M	£5,031,713	£5,081,654	£5,081,654	£5,080,944	£5,080,541
		M for D SUMDE	£200K	£82,867	£86,655	£86,655	£64,553	£59,058
		% of total of SUMDE	5.0%	2.0%	2.0%	2.0%	1.0%	1.0%
